# Letter from Berlin

**Published:** 1861-05

**Authors:** 


### SELECTIONS.

### *Letter from Berlin*

[Correspondence of the Maryland and Virginia Medical Journal.]

Berlin, January 24, 1861.

*Messrs. Editors:*—In conformity with your request, I shall now communicate to you some of my observations in the medical sphere of Berlin. I have been here during the greater part of two sessions of the University, and having availed myself of the superior clinical facilities which this place affords, in the various branches of the profession, I am led to believe that I shall be able to furnish you with some interesting facts.

I have found that the medical luminaries here are zealously devoted to the cultivation of the science, on the basis which distinguishes medicine of the present day from that of former times. In regarding nothing as reliable which does not emanate from ample observation, scientific research and rational deductions, they have been enabled to free it from many of the false notions which had gained a stronghold by tradition, and to contribute a great deal of valuable material to its reconstruction.

There are three hospitals connected with the University of Berlin—the Charité, (add to which the famous Pathological Institute), the Königliche Klinik der Universität, and the Obstetric Hospital. I need not announce the names of Virchow, Græfe, Frerichs, Traube, Langenbeck, Jüngken, Bærensprung and Martin, in connection with the institutions I have named, to convey to you an idea of their character. The Charité is a large and magnificent edifice, in which the following clinical lectures take place daily:—Two medical clinics, the one by Frerichs, the other by Traube; a surgical clinic by Jüngken; a clinic for venereal diseases and diseases of the skin by Bærensprung; a gynecological clinic by Martin, and a clinic for diseases of children by Elbert. Langenbeck conducts a surgical clinic at the Königliche Klinik der Universität, and Romberg has a clinic on the diseases of the nervous system in the same institution. Martin delivers clinical lectures at the Obstetric Hospital three times a week.

I think I will best meet the object of this correspondence by writing what I think may interest you in relation to the names I have mentioned. I shall begin with Dr. Albrecht von Græfe, Extraordinary Professor in the University. He has for many years diligently applied his genius to the study of opthalmology, and has thereby contributed much to the progress which this branch of science has made. Among the many great merits which may be claimed for him, the one standing paramount is his satisfactory description of glaucoma, the explanation of its symptoms, and what is more than all, the treatment which he has proposed for it, and successfully practised. He has proven beyond a doubt that the phenomena which this affection produces, depend on a preternatural intra-ocular pressure. He has called attention to the tense condition of the globe of the eye in support of this view, and has demonstrated with the opthalmoscope the pulsation of the arteries of the retina, which never becomes sensible to sight under any other condition.

As Græfe's highly valuable contributions on this subject have, as I believe, not yet appeared in English literature, you will, perhaps, indulge a few sketches from my notes in connection therewith. The disease being met with in different stages presents various forms. It may, for the sake of convenience, be divided into an acute and chronic form.

*Acute Glaucoma.*—The patient is suddenly attacked by a violent supra-orbital neuralgia, and almost invariably notices a blue ring around the flame of the lamp, when employing the eye in artificial light. Græfe thinks that this symptom may be considered pathognomonic of the affection. Sometimes all the colors of the rainbow are represented in this ring, yet in these cases the blue almost always seems to predominate. Occasionally there are cases met with where the patient sees two rings, the one within the other. When the eye is examined in this stage, it will be found bathed in tears, with the conjunctiva in a state of active congestion, the aqueous humour rendered cloudy, the pupil dilated and the iris pressed forward, thereby encroaching upon the dimensions of the anterior chamber. There is no other inflammatory condition of the eye which produces a dilation of the pupil, with the exception of one form of iritis, termed here, iritis serosa; but here the dilatation is of a less degree, while the iris is not pressed forward. The gravest symptom presented by glaucoma is the effect which it produces on the function of vision. Oftentimes the patient is rendered totally blind within an hour after the attack, while at others, this result takes place gradually. This fact is important in the differential diagnosis between glaucoma and iritis serosa; in the latter, vision does either not suffer at all, or which is oftener the case, only to a degree which can be explained by the cloudiness of the aqueous humour with which it is attended.

*Chronic Glaucoma.*—Acute glaucoma is of short duration, and gradually assumes the chronic form, if not prevented doing so by remedial measures, by the inflamatory symptoms receding. The supra-orbital pain disappears and the conjuctiva is relieved from its former condition, while the veins thereof may be seen to become enlarged. The cornea becomes very torpid, so that it may be touched with pointed paper, (a mode in which the sensibility of the cornea is tested here) without reacting. The pupil and iris remain in the same condition as in the acute form; while the power of vision is either greatly reduced or entirely abolished; to this is added the characteristic sea-green color which is reflected from the pupil. The continuance of this process compromises to a considerable degree the nutrition of the eye, and often produces a glaucomatous cataract. This must not be confounded with the sea-green color with which glaucoma is attended. Græfe thinks that this sea-green color is due to the combined effects of a dilated pupil, a cloudy aqueous humour, and the slight coloration which is met with in the lens of those of maturer age.

Græfe has proven that glaucoma is due to an inflammation in the deep tissues of the eye, in which the choroidea probably participates to the greatest extent. The process is attended with a hypersecretion or an exudation of fluid, so increasing the intra-ocular pressure, as to exert a paralyzing effect on the retina. The effect of pressure on the retina can be demonstrated by pressing the finger on the globe for a while, by which experiment we can subject ourselves to a temporary blindness. The tense condition of the globe of the eye in glaucoma, testifies in favor of Græfe's opinion; it can be constituted by placing a finger on each globe, and comparing the different degree of resistance which they offer. Another circumstance which points to an increased intra-ocular pressure is, the enlarged condition of the conjunctival veins; the blood meeting an obstacle in its return by pressure from within, seeks a passage through the veins of the conjunctiva. When the refractory mediums are not too much clouded, the arteries issuing from the papilla optici, will be seen to pulsate on subjecting the eye to an opthalmoscopic examination. That this phenomenon is caused by increased pressure, is proven by the fact, that it occurs in no other condition, and that it may be produced by causing pressure on the globe while examining with the opthalmoscope.

*Treatment.*

Having satisfactorily explained the nature of the disease, Græfe resorted first to the various antiphogistic remedies, and then to paracentesis of the cornea, but found these inadequate to control it. Having noticed that excision of a piece of the iris, in pursuing other indications, greatly reduced intra-ocular pressure, he tested its efficacy in glaucoma and found it fraught with the happiest results. The oculists of Vienna, Paris and London, have all adopted Græfe's mode of treatment. If iridectomy be performed in the acute stage, a perfect restoration of sight may be reckoned on. I have seen patients who have been enabled to read the finest print a short while after the operation, though they had been blind for years before. It is however, evident from the nature of the disease, that the prognosis depends a good deal on the time it had existed. Though the disease may be very chronic, if the patient can yet discern light, the operation may be performed with a good deal of certainty that vision will be restored to a considerable degree. It is necessary to add that the disease sometimes deviates from the description I have given.

Græfe has seen a few cases where progress was so rapid, that though he performed the operation within seven or eight days after the attack, it has proved unavailing, so great was the damage inflicted on the retina. It is therefore important, that the operation be performed as soon as the diagnosis is made. Surgeons must not permit themselves to be intimidated by the violence of the inflammatory symptoms. I have seen patients, the vessels of whose conjunctiva were injected to a maximum degree, and who have been unable to sleep for nights in succession from the intense supra-orbital pain, relieved to considerable degree of these symptoms, and sink into a quiet slumber a short while after the operation. The more violent the symptoms are, the greater is the indication. Græfe justly places this operation in the same category with tracheotomy, which every physician should be prepared to perform at a moment's warning. In some rare cases, the blue ring spoken of, the supra-orbital pain, the dilated pupil and slight disturbance of vision, act as prodromic symptoms; after remaining for a short time they disappear and re-appear after a longer or shorter interval with all the pernicious effects of the disease. In some of these cases where the symptoms reach but a minimum degree, it is not absolutely necessary to resort to the operation immediately, especially when the patient is watched by the physician; but as the operation is a perfectly safe one when skillfully performed, it is better to resort to it when there is reason to believe that the patient may apply a second time for medical aid, and when it may be too late. Science has just reason to congratulate itself in now having a means within its grasp, to battle successfully against a disease which formerly it was forced to recognize as a perpetual sentence to blindness. It is all-important that the operation be performed in the correct manner. The operation consists in an excision of a piece of the iris in the shape of a V, the base of which corresponding to its pupilary margin, the apex to its periphery. The operator fixes the eye with a pair of forceps, holding a fold of conjunctiva; he then makes an incision through the cornea at its pheriphery or through the sclerotica, a little behind it; he then inserts through this incision a curved pair of forceps, grasps a piece of the iris and pulls it out; an assistant clips it off. The operator must avoid injuring the lens, with either the knife or forceps, otherwise he will inflict a traumatic cataract on the patient. With this view, the operator, when he feels that the point of his instrument (which is a lancet-shaped knife) has pierced the tunics, must give the handle a direction which will make the point proceed farther in a direction forwards, otherwise he will puncture the iris and thereby injure the lens. The curved forceps must be introduced with the blades closed, and the convexity towards the patient; when they have entered and reached a point near the pupil, the blades must be spread, a fold of the iris will insinuate itself between them, which must be grasped and gently withdrawn. If the forceps go beyond the pupil there is great danger of wounding the lens. The artificial pupil thus made must reach as near to the periphery of the iris as possible; this is especially important in chronic cases. Græfe therefore, enters the anterior chamber through an incision in the sclerotica; this can be done with facility, as the iris is not inserted at the juncture of the cornea with the sclerotica as most anatomical works have it, but a little behind it. Though the efficacy of iridectomy in reducing intra-ocular pressure is a well established fact, the manner in which it produces this effect is difficult of explanation. Græfe has instituted various anatomical investigations with a view of solving this problem, and though he has offered various explanations, he has not yet satisfied himself. The explanation which bears the greatest degree of plausibility is, that the pressure which displaces the iris is concentrated behind it, and that the operation promotes its uniform diffusion within the globe.

Græfe has under his charge a fine hospital, the "Augen Klinik," devoted to the diseases of the eye exclusively. Here he delivers clinical lectures three times a week, each lasting two hours; in addition to this, he also delivers theoretical lectures on the diseases of the eye. He also conducts a practical course of operative ocular surgery, in which supplying his pupils with phantoms, eyes, rabbits, and subjects, he offers them every facility to qualify themselves in this branch. His assistants, Drs. Leibreich and Schweigger respectively, teach the application of the opthalmoscope, and the normal and pathological anatomy of the eye.

Græfe is in the prime of life, and devotes himself entirely to the interests of the science. He is engaged with Prof. Donders, of the Utrecht, and Prof, Arlt, of Vienna, in editing the "Archiv für Opthalmologie." The work has reached its sixth volume, and a volume is added to it annually.

Yours respectfully, A. F.

